# Supplementary material for: Reported Adverse Events in Patients with CF Receiving Treatment with Elexacaftor/Tezacaftor/Ivacaftor: 5 Years Observational Study
Source: J Clin Med. 2025 Jun 18;14(12):4335. doi: 10.3390/jcm14124335 (PMC12194673; doi:10.3390/jcm14124335)
Supplement: Supplementary file 1 [file jcm-14-04335-s001.zip › jcm-3630856-supplementary.pdf]

Supplementary data to:

Reported Adverse Events in Patients with CF Receiving Treatment with Elexacaftor/Tezacaftor/Ivacaftor: 5 Years Observational Study

Francesca Lucca, Ilaria Meneghelli, Gloria Tridello, Francesca Buniotto, Giulia Cucchetto, Sonia Volpi, Emily Pintani, Valentino Bezzerri, Marco Cipolli

Table S1. Modifications of ETI therapy in response to AEs, broken down by SOC. The frequency of the worst outcome is reported for each SOC.

Permanent discontinuation	Permanent dosage modification	Temporary interruption	Temporary dosage modification	None		
4/24 (16.7%)	11/24 (45.8%)	3/24 (12.5%)	3/24 (12.5%)	3/24 (12.5%)	24	Investigations
2/24 (8.3%)	11/24 (45.8%)		2/24 (8.3%)	9/24 (37.5%)	24	Psychiatric disorders
3/24 (12.5%)	1/24 (4.2%)	4/24 (16.7%)	3/24 (12.5%)	13/24 (54.2%)	24	Skin and subcutaneous tissue disorders
1/23 (4.3%)	8/23 (34.8%)	4/23 (17.4%)		10/23 (43.5%)	23	Gastrointestinal disorders
	6/13 (46.2%)		1/13 (7.7%)	6/13 (46.2%)	13	Musculoskeletal and connective tissue disorders
	5/10 (50.0%)		3/10 (30.0%)	2/10 (20.0%)	10	Nervous system disorders
4/9 (44.4%)	1/9 (11.1%)	2/9 (22.2%)	1/9 (11.1%)	1/9 (11.1%)	9	General disorders and administration site conditions
	1/8 (12.5%)		3/8 (37.5%)	4/8 (50.0%)	8	Respiratory, thoracic and mediastinal disorders
			2/4 (50.0%)	2/4 (50.0%)	4	Eye disorders
				1/1	1	Ear and labyrinth disorders
1/1					1	Hepatobiliary disorders
				1/1	1	Reproductive system and breast disorders
